# Supplementary material for: Active site specificity profiling datasets of matrix metalloproteinases (MMPs) 1, 2, 3, 7, 8, 9, 12, 13 and 14
Source: Data Brief. 2016 Feb 22;7:299–310. doi: 10.1016/j.dib.2016.02.036 (PMC4777984; doi:10.1016/j.dib.2016.02.036)
Supplement: Supplementary file 10 — Supplementary material [file mmc10.zip › WebPICS_hMMP8_T_1%/subsite_coop.html]

 

PICS results


- # About
- # Manual
- # Analysis
- # Results

- Redraw
- Seqlogo
- Dependency
- Coop

**Potential subsite cooperativity analysis for hMMP8\_T\_1%**   
Minimum difference for subsite dependency was set to +- 10 percentage-points  
Subsite dependency was checked for all positional occurences above 1 x natural abundance  
**Subsite dependency check is restricted to P3 - P3'**

|  |  |  |  |
| --- | --- | --- | --- |
| Fixed residue | Affected residue(s) | Change (percentage-points) | Vice-Versa change |
| P3\_A | P2\_L | 12.1 | 10.4 |
| P3\_P | P2\_E | 10.4 | 24.5 |
| P3\_T | P2prime\_H | 12.2 | 19.3 |
| P3\_V | P2\_S | 10.1 | 12.3 |
| P3\_V | P3prime\_S | 10.8 | 10.1 |
| P2\_D | P2prime\_I | 18.2 | 14.5 |
| P2\_E | P1\_Q | 14.7 | 21.3 |
| P2\_E | P1prime\_Y | 10.6 | 14.0 |
| P2\_F | P2prime\_W | 15.6 | 59.8 |
| P2\_L | P1\_N | -10.2 | -12.8 |
| P2\_M | P2prime\_M | 16.8 | 10.4 |
| P2\_Q | P1prime\_V | 12.6 | 10.7 |
| P2\_W | P2prime\_T | 35.7 | 10.4 |
| P2\_Y | P2prime\_Q | 10.7 | 12.8 |
| P1\_G | P1prime\_W | 11.2 | 48.1 |
| P1prime\_C | P2prime\_H | 14.0 | 19.9 |
| P1prime\_I | P2prime\_L | 16.7 | 14.5 |

  
**PICS analysis of protease: hMMP8\_T\_1%**  
335 cleavage sites analyzed  
PICS library made with (T)rypsin, (G)luC or (C)hymotrypsin:   
Cutoff for graphic display: 2 x natural abundance  

|  |  |
| --- | --- |
| Positional occurences  (table for total and relative (in %) values) | Occurences relative to natural abundance (table) |
|  |  |
